# Contexts of Anointing Behavior in a Group of Blond Capuchin Monkeys (*Sapajus flavius*) Inhabiting an Atlantic Forest Fragment

**DOI:** 10.1002/ajp.70119

**Published:** 2026-01-26

**Authors:** Ana Paula de Brito‐Araújo, Natsumi Hamada‐Fearnside, Simone Peruzzo, Italo Ferreira Pereira, Poliana Gabriele Alves de Souza Lins, Kyle Miller, Patrícia Elesbão da Silva Rodrigues, Luiz Felipe Moretti Iniesta, Renata Gonçalves Ferreira

**Affiliations:** ^1^ Department of Physiology and Behavior Federal University of Rio Grande do Norte Natal Rio Grande do Norte Brazil; ^2^ Postgraduate Program in Ecology and Biodiversidade Federal University of Mato Grosso Cuiabá Mato Grosso Brazil; ^3^ Department of Geography, Planning and Environment Concordia University Montreal Quebec Canada; ^4^ Butantan Institute São Paulo Brazil

**Keywords:** fur‐rubbing, self‐medication, social behavior, benzoquinone, auto‐medicação, fur‐rubbing, social behavior, benzoquinona

## Abstract

Parasitism is one of the primary causes of biotic stress in several taxa, and behaviors resembling self‐medication have been documented in numerous species. Anointing involves the application of chemical substances derived from animals, plants, mud, soils, and minerals, often emitting a pungent odor, onto an animal's body. We examined the circumstances surrounding 34 bouts of anointing with millipedes in a group of blond capuchin monkeys (*Sapajus flavius*) inhabiting an Atlantic Forest fragment in northeastern Brazil. Over 412 h of observation, we collected behavioral data through photographs, video recordings, and ad libitum field notes. We collected and identified the millipedes to the species level. We tested three non‐mutually exclusive hypotheses on the function(s) of this behavior: self‐medication, social bonding, and opportunistic use. We analyzed data in R using nonparametric tests due to low sample size. Anointing occurred at a rate of 8 episodes every 100 h. Millipedes used during anointing are from species that produce benzoquinone. The behavior was more frequently observed in the inferior strata, during midday, while the group is mostly foraging, in periods of higher rainfall, when millipedes emerge from the ground, and during the high fruit productivity season, when the capuchins' activity budget is less constrained. Although anointing occurred at similar rates in solitary and social contexts, adult males were more actively engaged in anointing bouts, indicating sex difference in this behavior, and a possible social function. The observed pattern suggests anointing is a multifunctional behavior, combining elements of self‐medication, opportunity, and social interaction.

## Summary

- We recorded 34 bouts of anointing with millipedes by capuchin monkeys at a rate of 0.08 bouts per contact hour.
- Similar rates of solitary and social anointing, but adult males were more engaged in bouts.
- Anointing is a multifunctional behavior, combining elements of self‐medication, opportunity, and social interaction.

## Graphical abstract

Wild blond capuchin anoints with millipede species that secrete a chemical capable of repelling ectoparasites, suggesting a self‐medicative function. Social and solitary anointing bouts occurred at similar frequencies, but with greater engagement by adult males, indicating that anointing is a multifunctional behavior.

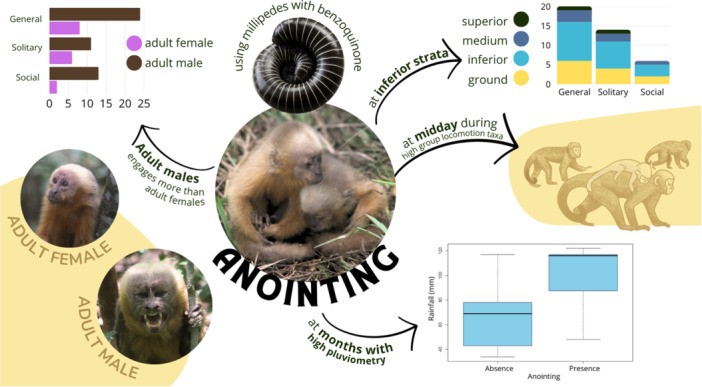

## Resumo

O parasitismo é uma das principais causas de estresse biótico em diversos táxons, e comportamentos semelhantes à automedicação são documentos em várias espécies. A unção consiste na aplicação de substâncias químicas derivadas de animais, plantas, lama, solos e minerais, frequentemente emitindo um odor forte, sobre o corpo do animal. Examinamos as circunstâncias em torno de 34 episódios de unção com milípedes em um grupo de macacos‐prego‐galego (*Sapajus flavius*) que habitam um fragmento de Mata Atlântica no nordeste brasileiro. Ao longo de 412 horas de observação, coletamos dados comportamentais por meio de fotografias, gravações de vídeo e notas de campo ad libitum. Coletamos e identificamos os milípedes ao nível de espécie. Testamos três hipóteses não mutuamente exclusivas sobre possíveis funções desse comportamento: automedicação, vínculo social e uso oportunista. Analisamos os dados no R utilizando testes não paramétricos devido ao tamanho amostral reduzido. A unção ocorreu a uma taxa de 8 episódios a cada 100 horas. Os milípedes usados durante as unções são de espécies que excretam benzoquinona. O comportamento foi mais frequentemente observado nos estratos inferiores da vegetação, ao meio‐dia, enquanto o grupo estava principalmente forrageando, em períodos de maior precipitação, quando os milípedes emergem do solo, e durante a estação de alta produtividade de frutos, quando o orçamento de atividades dos macacos é menos restrito. Embora a unção tenha ocorrido em taxas semelhantes em contextos solitários e sociais, os machos adultos estiveram mais ativamente engajados em episódios de unção, indicando uma diferença sexual nesse comportamento e uma possível função social. O padrão observado sugere que a unção é um comportamento multifuncional, combinando elementos de automedicação, oportunismo e interação social.

## 1 Introduction

Anointing is a behavior characterized by the application of chemical substances derived from animals and plants, including mud, onto an animal's body (Baker 1996; Messer et al. 2022; Weldon 2004). Birds and mammals are the two groups most extensively documented in the literature for practicing anointing (Morozov 2015). Anointing in birds has been identified as a strategy to combat ectoparasites, both on their bodies and in their nests (Clayton et al. 2010; de Roode et al. 2013). This behavior has been described in more than 200 species of Passeriformes birds (Morozov 2015) and is referred to as anting (anointing with ants and other arthropods), varying between passive anting or active anting (Craig 1999; Morozov 2015; Ohkawara et al. 2022; Coulson 2023; Perin Marcon and Andriola 2023).

Anointing is also reported in many mammals. There is a description of hedgehogs (*Atelerix pruneri*) chewing toads and spreading their saliva mixed with toad skin secretion onto their spines using their tongues (Brodie 1977). Similarly, wild rats (*Rattus rattoides*) display this behavior by manipulating the anal secretions of Siberian weasels (*Mustela sibirica*) and smearing the anal secretions onto their bodies (Xu et al. 1995). Results from Xu et al. (1995) and Brodie (1977) demonstrated that anointing is innate in both *A. pruneri* and *R. rattoides*, as it was observed in laboratory conditions in individuals just a few days old, indicating that the expression of this behavior does not require prior learning.

Primates have been found to engage in anointing by manipulating herbs by *Pongo pygmaeus* (Morrogh‐Bernard 2008), leaves by *Pongo abelii* (Laumer et al. 2024), plants and millipedes by *Aotus* spp. (Zito et al. 2003; Jefferson et al. 2014), leaves by *Ateles geoffroyi* (Campbell 2000; Laska et al. 2007), plants, ants, and millipedes by *Cebus* sp. and *Sapajus* sp. (Alfaro et al. 2011; Verderane et al. 2007; Medeiros et al. 2020); tree exudates by *Leontopithecus chrysomelas* (Guidorizzi and Raboy 2009), millipedes by *Eulemur* sp. (Birkinshaw 1999; Peckre et al. 2018).

Anointing has been observed in both solitary or social contexts, and four nonmutually exclusive functions have been proposed to explain this behavior: (1) self‐medication (as an ectoparasite repellent, antiseptic, anti‐inflammatory remedy, or anesthetic), which is the most supported hypothesis in the literature (Baker 1996; Falótico et al. 2007; Verderane et al. 2007; Paukner and Suomi 2012; Bowler et al. 2015; Medeiros et al. 2020; Messer et al. 2022); (2) scent marking, related to reproductive regulation (Bowler et al. 2015); (3) social bonding, similar to grooming or mutual application (Verderane et al. 2007; Paukner and Suomi 2012; Bowler et al. 2015; Messer et al. 2022); and (4) opportunistic and recreational or amusement purposes, with some reports indicating a state of frenetic trance observed in animals during anointing (Birkinshaw 1999; Baker 1996; Verderane et al. 2007; Bowler et al. 2015; Medeiros et al. 2020).

Capuchin monkeys are renowned for their manipulative skills, social tolerance, and diverse behavioral repertoire, enabling them to inhabit a wide range of environments, including tropical forests such as the Amazon and Atlantic Forests, savannah environments like the *Cerrado*, and seasonally dry forests such as the *Caatinga* and flooded areas like mangroves throughout Central and South America (Fragaszy et al. 2004). Anointing behavior has been documented in both genera of capuchin monkeys, *Cebus* and *Sapajus*, in wild and captive conditions (see Alfaro et al. 2011 for a review). In capuchin monkeys, anointing is commonly associated with the manipulation of arthropods (Alfaro et al. 2011; Medeiros et al. 2020; Valderrama et al. 2000; Verderane et al. 2007), fruits (Baker 1996; Paukner and Suomi 2008; Bowler et al. 2015), leaves (Alfaro et al. 2011), and anthropogenic products, such as insect repellent (*Cebus capucinus*, Santos et al. 2019). This behavior appears to be more frequent in *Cebus* spp., which also utilize a greater variety of plant species. *Sapajus* spp., and have been found to use a broader range of arthropods, including ants and millipedes (Alfaro et al. 2011). There are three potential functions of anointing that have been proposed for capuchin monkeys: (1) self‐medication (Baker 1996; Falótico et al. 2007; Paukner and Suomi 2012; Bowler et al. 2015; Medeiros et al. 2020; Messer et al. 2022); (2) social functions (Verderane et al. 2007; Paukner and Suomi 2012; Bowler et al. 2015; Messer et al. 2022); and (3) an opportunistic (Baker 1996; Verderane et al. 2007; Bowler et al. 2015; Medeiros et al. 2020).

Baker's (1996) study on free‐ranging *C. capucinus* described anointing behavior as self‐medication, with an adaptive function related to skin protection or parasite regulation, taking advantage of the bioactive compounds present in the plants. However, a recreational function was also suggested, as anointing was observed during foraging for fruits or pods, with monkeys often frantically rubbing these on their fur and salivating. The opportunity/recreational function was suggested since the author also noted a correlation between high anointing rates and rainy periods. Millipedes are burrowing arthropods that spend most of their lives underground, becoming more active during wet seasons and surfacing when the soil becomes moist (Ashwini and Sridhar 2006; Crawford et al. 1987; Dangerfield and Telford 1991; Dangerfield 1998).

Ants and millipedes are among the arthropods most commonly observed being used for anointing by capuchin monkeys (Alfaro et al. 2012). They can be used as a form of self‐medication, since the pungent smell of the compounds secreted by these arthropods has been suggested to function as an ectoparasite repellent. Verderane et al. (2007) and Falótico et al. (2007) documented the use of carpenter ants (*Camponotus rufipes*) during anointing bouts by semi‐free‐living *S. apella* at Parque Ecológico do Tietê, São Paulo, Brazil. Verderane et al. (2007) reported that this behavior was performed equally across all sexes and age classes, suggesting that it potentially protects against ectoparasites. Similarly, Valderrama et al. (2000) described anointing in a free‐living group of *Cebus olivaceus*, using millipedes (*Orthoporus dorsovittatus*) and discussed how the chemical secretions released by these arthropods, rich in benzoquinone, may function as insect repellents.

In a descriptive study on capuchin monkeys (*Sapajus flavius*) in an Atlantic Forest fragment, Medeiros et al. (2020) observed anointing behavior with millipedes (Spirobolida: Rhinocricidae), which secrete a chemical compound rich in benzoquinone that has repellent properties against hematophagous arthropods. The three anointing bouts recorded in this study occurred during the wet season, which the authors related to a potential response to the heightened presence of disease‐carrying insects during this period. In their study, anointing was observed in 13 individuals of varying age and sex classifications, both in solitary contexts (1 bout with 1 event) and social contexts (2 bouts with 10 events), suggesting both individual and group‐level responses to environmental pressures.

The social function of anointing behavior in capuchin monkeys remains a subject of debate. Paukner and Suomi (2012) described increased agonistic and reduced affiliative behaviors in a captive group of *Sapajus* (formerly *Cebus*) *apella*, following anointing events involving onions and apples. The dominant individuals exhibited heightened aggression towards those individuals carrying the scent of the manipulated substances, potentially reinforcing monopolization of resource access. Conversely, Bowler et al. (2015) reported a positive social function with potential medicinal benefits in captive *Sapajus libidinosus*. They suggested that areas of the body anointed during social bouts might be inaccessible when self‐anointing. Furthermore, their investigation revealed no significant differences in anointing behavior between sex‐age classes.

Messer et al. (2022) described five sociograms for two captive groups, denoted “West” and “East,” of *S. apella*. These sociograms were constructed based on indices of association between individuals during five distinct conditions: no resource present (baseline), during and after anointing with scarce resources, and during and after anointing with abundant resources. The patterns of proximity between individuals exhibited variations contingent on the type and density of the available resource (onion) for the anointing. The “West” group displayed an increase in cohesion after anointing, regardless of the resource density, thereby supporting the hypothesis of social bonding facilitated by anointing. Conversely, the “East” group manifested cohesion solely after anointing when the resource was scarce, probably due to limited access.

Studies on hormonal changes associated with fur‐rubbing behavior in captive *S. apella* show that oxytocin levels and social behavior vary nonlinearly. Benítez et al. (2018) demonstrated that oxytocin increases similarly after grooming and fur‐rubbing bouts, with no age or sex differences. In the studied group, fur‐rubbing increased affiliative behaviors towards preferred partners but did not have an overall prosocial effect towards less‐affiliated group mates. Furthermore, while the increase in oxytocin lasted for at least 60 min, affiliative behaviors decreased from 30 to 45 min after the fur‐rubbing bout. The authors attribute this decrease to an overall anxiolytic (calming) effect mediated by oxytocin, which could derive from the fur‐rubbing itself or from the body contact during the bout. Thus, while hormonal evidence supports a social explanation, it also highlights the complexity of these interactions.

In this study, we analyzed the context of 34 bouts of millipede anointing observed in a group of blond capuchin monkeys (*S. flavius*) within an Atlantic Forest fragment in Northeastern Brazil. Based on previous research and considering self‐medication, social bonding, and opportunity functions that have been suggested for anointing, we formulated the following predictions. Regarding the self‐medication function: (1.1) we expect that the millipedes used in anointing release chemicals possessing repellent or anesthetic properties, which could be employed for self‐medication; and (1.2) there will be no difference between age and sex classes, as seen in Laska et al. (2007) and in Verderane et al. (2007), as long as adult and juveniles, adult males and females have the same health needs to protect themselves against ectoparasites. Concerning the social function: (2.1) we expect there will be higher rates of social anointing compared to solitary anointing, as social anointing may facilitate affiliative interactions. Regarding opportunistic functions: (3.1) we predict that anointing behavior will occur at higher rates on the ground and near/on the edge, where millipedes are more readily found out of the leaf litter‐free edge where the visibility of arthropods is higher, compared to the leaf litter area within the fragment and other strata; (3.2) an increased occurrence of anointing behavior during midday, when locomotion and foraging activity within the group are at their peak; (3.3) during months of high arthropod abundance (ants, scorpions, millipedes, spiders, and beetles), the occurrence of anointing using millipedes will be more frequent; and (3.4) a positive correlation between anointing and high pluviometry (wet season) because millipedes emerge from the ground more often.

## 2 Material and Methods

### 2.1 Study Population

The blond capuchin monkey group observed in this study inhabits a U‐shaped Atlantic Forest fragment, with one side located within the state of Paraíba and the other within the state of Pernambuco, in Northeastern Brazil (07°31′34.2″S 034°57′47.7″W). The study site consists of a secondary Atlantic Forest fragment spanning 270 ha, which was surrounded by industrial sugarcane plantations and, more recently, by agricultural settlements. A maximum of 134 individuals, covering an area of ~90 ha, were counted in the Paraíba area group, although they split daily into groups of varying compositions (personal observation). We regularly follow one group of about 60 individuals with an age–sex distribution of 45% adult males, 23% females, and 32% (Peruzzo 2023). The food productivity of this fragment has been continuously monitored since 2014, via fruit and invertebrate traps in 30 sampling areas (Pereira 2024). This area presents a relatively low fruit offer throughout the years, in comparison to other fragments used by capuchin monkeys: yielding less than 288 kg of fruits per hectare and 1.132 kg of insects/arthropods per hectare (Izar et al. 2018).

### 2.2 Data Collection

Typically, researchers tracked the group during daylight hours, recording behavior and GPS locations, occasionally using photo and video cameras. We used the definition proposed by Weldon (2004) of anointing behavior, which is when an individual rubs with its hands or mouth the substances from heterospecifics on its own or another's body. Given that anointing with plant matter has never been recorded in this population, our study focused exclusively on behaviors involving millipedes. Millipede remnants from anointing bouts were photographed and served as a guide for collecting live male specimens, required for species identification. The collected individuals were dissected and identified by specialists in Diplopoda by analysis of photographs of male reproductive organs. Data were collected from April 2021 to June 2022.

Each occurrence of anointing behavior was systematically named, analyzed, and classified as follows: *solitary*, signifying instances in which only one individual engaged in the behavior in isolation, or *social*, designating situations in which two or more individuals engaged in anointing, either in close body contact or in cases involving a bout with two or more groups of individuals performing anointing within a maximum distance of 10 m. In the latter scenario, our analyses did not consider each of the groups as distinct social events but rather treated all individuals within 10 m as a unique social bout. The individuals' age–sex classes were determined by evaluation of body size and coat characteristics, following the criteria established by Izawa (1980), with no individual identification. In our data, observations were collected from adult males and females, as well as juveniles; no infants were observed engaging in anointing bouts.

**Figure 1:**
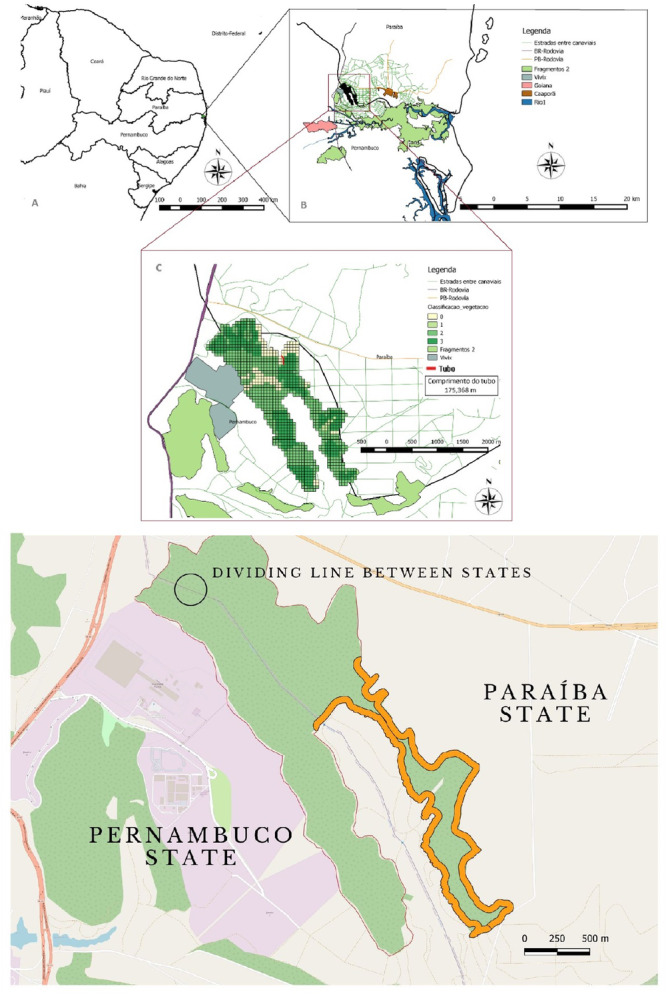
Graphical representation of the studied Atlantic Forest fragment, surrounded by sugar cane crops. Highlighted in orange is the extent of the fragment's edge inward by 50 m.

We classified the strata based on height as follows: ground (0–29 cm), inferior (30 cm–5 m), medium (5–15 m), and superior (15 m above). To examine temporal patterns, we categorized the hours of the day into three distinct periods: morning (7:00 to 9:59 a.m.), midday (10:00 a.m. to 1:59 p.m.), and afternoon (2:00 to 4:59 p.m.). Furthermore, we identified bouts occurring within 50 m from the edge of the fragment as “edge,” while those occurring more meters inside the fragment were designated as “interior” (see Figure 1).

Precipitation data were acquired from the official TABU distillery data, which provides rainfall data for the area of the distillery, located about 11 km from the studied forest fragment. Since 2014, monthly food productivity has been monitored across 30 sampling circular areas of 10 m diameter, corresponding to 1% of the overall study area—90 ha. In each sampling area, we measured the phenology of all trees with DBH over 12.5 cm using the Fournier method for fruits, and also considering the weight of fruits, insects/arthropods collected in traps on the ground (see Lins and Ferreira 2019 for a detailed description of the food productivity methodology).

### 2.3 Data Analyses

Statistical analyses were conducted using R 4.2.2 (R Core Team 2022). To test hypotheses regarding differences in the occurrence of anointing behavior with millipedes among age (adult vs. juveniles), sex (adult males vs. adult females) classes, strata (ground, inferior, medium and superior), and areas (edge of the fragment vs. interior of the fragment), we employed chi‐square analysis, with expected values based on the number of hours of observation of each age class, sex, strata, and area. A binomial test was employed to test the difference between social and solitary conditions. Additionally, we utilized a Mann–Whitney test to compare the average values of rainfall and food yield between months with and without anointing records (presence/absence), and a Spearman test to verify correlation between rainfall and fruit abundance. We used a type one alpha error < 0.05; however, given the increase in type two error due to low sample size, we will also discuss the trends in significance until alpha < 0.055. Table 1 provides a characterization of the 34 anointing bouts, and Table 2 presents the predictions and statistical analyses employed to test the hypotheses. As individuals in the group are not identified, it is possible that the same individual was present in different bouts; however, for the present analyses, we assumed independence of data.

**Table 1:** Characteristics of observed social anointing events.

Date	Pluviometry (mm)	Bout context	Individuals involved	Strata	Edge versus interior	Start time	Duration (s)
April 3, 2021	116	Solitary	AF^1^	4	—	10:01	75
April 6, 2021	116	Solitary	J^1^	3	—	11:03	46
May 2, 2021	117	Solitary	AM^1^	—	E	15:57	459
May 8, 2021	117	Solitary	J^1^	3	IN	14:24	111
May 16, 2021	117	Social	AM^2^	3	—	09:37	216
May 17, 2021	117	Social	AF^1^ + AM^1^	—	E	11:43	—
May 27, 2021	117	Solitary	AM^1^	—	E	11:54	—
June 19, 2021	122	Social	J^1^ + AM^1^	1	IN	10:29	109
June 21, 2021	122	Social	AM^2^	2	E	10:24	219
June 26, 2021	122	Social	AF^1^ + J^1^	—	E	11:46	—
June 26, 2021	122	Solitary	AF^1^	—	E	12:00	—
June 26, 2021	122	Solitary	AF^1^	—	E	13:02	36
June 28, 2021	122	Solitary	AM^1^	—	E	14:53	—
July 15, 2021	117	Social	AM^2^	1	—	11:50	604
July 15, 2021	117	Social	J^3^	1	—	12:25	—
July 15, 2021	117	Social	AM^2^ + J^2^	1	—	13:27	—
July 15, 2021	117	Social	AM^1^ + J^1^	2	E	14:20	65
September 30, 2021	48	Solitary	AM^1^	_	IN	08:55	—
January 28, 2022	68	Solitary	AM^1^	3	IN	08:52	—
January 28, 2022	68	Solitary	AM^1^	2	IN	10:13	—
January 28, 2022	68	Solitary	J^1^	1	E	10:57	—
January 31, 2022	68	Solitary	AM^1^	1	E	14:27	—
February 18, 2022	78	Solitary	J^1^	2	E	15:04	—
February 20, 2022	78	Solitary	J^1^	2	IN	14:01	—
March 11, 2022	97	Social	J^2^	1	E	14:12	—
March 11, 2022	97	Solitary	AM^1^	2	E	14:48	—
March 19, 2022	97	Solitary	AF^1^	2	E	14:19	—
April 15, 2022	116	Solitary	AM^1^	2	E	09:34	—
April 27, 2022	116	Social	AM^1^ + J^1^	2	IN	10:19	—
May 1, 2022	117	Social	AM^1^ + J^1^	—	—	—	—
May 16, 2022	117	Solitary	AF^1^	2	E	07:34	—
June 10, 2022	122	Solitary	AF^1^	1	E	11:20	—
June 10, 2022	122	Solitary	AM^1^	1	E	11:22	—
a	—	Solitary	AM^1^	—	—	—	—

*Note:* Listed are the date of observation, average monthly rainfall, whether the anointing bout was solitary or social, and the individuals involved in the behavior. ^1, 2, 3^ represent the number of individuals in each category (e.g., AM^1^ is one adult male); the strata on which the bout occurred (1) ground; (2) inferior = 0.3–5 m; (3) medium = 5.1–10 m; (4) superior > 15 m; whether the event happened within 50 m of the edge (E) or further than 50 m or interior (IN); the time the event was registered; and the duration of the bout in seconds, when possible.

Abbreviations: AF, adult female; AM, adult male; J, juvenile; –, missing information.

^a^ Data from a video of an adult male carrying an infant that was performing a bout of solitary anointing; however, due to the loss of its metadata, the date, strata, and location could not be established.

**Table 2:** Variables tested, number of recorded bouts under each variable, and total number of observed individuals (*n*).

Test variables	*N* bouts (category)				Total *N*	*χ* ^2^, MW or *R*s	df	*p*	Statistical test
Social versus solitary (freq)	12 (SC)	22 (SL)			34			0.121	Binomial
Social versus solitary (dur)	c					7		0.313	Mann–Whitney
Female–male (all)	24 (AM)	8 (AF)			32	23.97	1	< 0.001	Chi‐square^a^
Female–male (solitary)	11 (AM)	6 (AF)			17	7.14	1	< 0.001	Chi‐square^a^
Female–male (social)	13 (AM)	2 (AF)			15	18.54	1	< 0.001	Chi‐square^a^
Juvenile–adult (all)	17 (J)	32 (A)			49	0.24	1	0.619	Chi‐square
Juvenile–adult (solitary)	5 (J)	17 (A)			22	0.77	1	0.381	Chi‐square
Juvenile–adult (social)	12 (J)	15 (A)			27	2.13	1	0.144	Chi‐square
Strata (all)	6 (G)	10 (I)	3 (M)	1 (S)	20	11.61	3	0.008	Chi‐square^a^
Strata (solitary)	4 (G)	7(I)	2 (M)	1 (S)	14	7.17	3	0.054	Chi‐square^b^
Strata (social)	2 (G)	3 (I)	1 (M)	0 (S)	6	4.61	3	0.159	Chi‐square
Edge versus interior (all)	19 (E)	7 (IF)			26	0.015	1	0.902	Chi‐square
Edge versus interior (solitary)	14 (E)	5 (IF)			19	0.001	1	0.975	Chi‐square
Edge versus interior (social)	5 (E)	2 (IF)			7	0.001	1	0.973	Chi‐square
Day period (all)	5 (1)	24 (2)	0 (3)		32	22.94	2	< 0.001	Chi‐square^a^
Day period (solitary)	4 (1)	14 (2)	3 (3)		21	9.51	2	0.009	Chi‐square^a^
Day period (social)	1 (1)	10 (2)	0 (3)		11	15.29	2	< 0.001	Chi‐square^a^
Pluviometry versus fruit offer	c					0.54		0.034	Spearman^a^
Fruit offer (monthly)	c					−2.11	14	0.053	Mann–Whitney b
Insects offer (monthly)		c				−0.85	14	0.409	Mann–Whitney
Rainfall (monthly)		c				−2.69	14	0.017	Mann–Whitney^b^

*Note:* The last column indicates the statistical test used, considering a significance level of 0.05.

^a^ Significant result (*p* < 0.05).

^b^ Significant result following alpha error < 0.055; SC (social); SL (solitary); (E) occurrence of anointing from 0 to 50 m from the edge of the fragment; (IN) occurrence of anointing more than 50 m from the edge/interior; (AM) adult male; (AF) adult female; (J) juvenile; (G) ground strata; (I) inferior strata (0.3–5 m); (M) medium strata (5–15 m); (S) superior strata (above 15 m); (1) period from 7:00 to 9:59 a.m.; (2) period from 10:00 a.m. to 12:59 p.m.; (3) period from 1:00 to 3:59 p.m.).

^c^ Continuous variables; chi‐square (*χ* ^2^), Mann–Whitney value (*U*) or Spearman test values (rho); df = degrees of freedom; *p* value, statistical test used.

This study follows the American Society of Primatologists' Principles for the Ethical Treatment of Non‐Human Primates and the Code for Best Practices in Field Primatology. This project was approved by the Universidade Federal do Rio Grande do Norte's bioethics committee (Approval Number: 251.014/2021 and SISBIO 76835‐3).

## 3 Results

The activity budget of the *S. flavius* group in this period was composed of (% mean ± SD): locomotion (22 ± 20), foraging (20 ± 27), social (mean 23 ± 38), ingestion (12 ± 20), sexual (9 ± 2), rest (7 ± 7), and other behaviors (7%) (Araújo 2022). Over 412 contact hours, we observed 34 anointing bouts involving millipedes, corresponding to a rate of 0.08 bouts per contact hour. We recorded the onset time of the anointing behavior in 32 out of 34 bouts and obtained GPS coordinates (using LocusMap Pro, version 4) for 26 of these, the strata (height above the ground) in 20 bouts, and we collected a total of 16.2 min of video footage documenting the anointing behavior.

**Figure 2:**
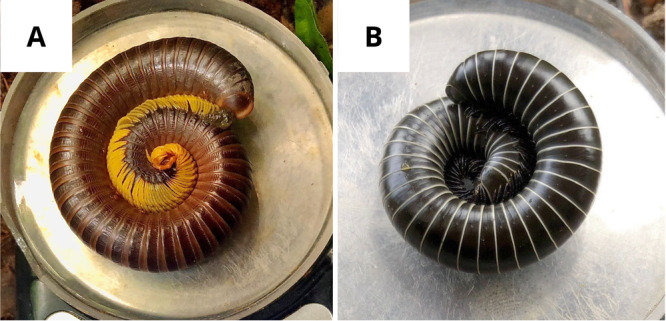
Millipedes utilized in anointing behaviors. (A) Cladostreptus angustifrons (Spirostreptida, Spirostreptidae). (B) *Rhinocricus birivus* (Spirobolida, Rhinocricidae).

**Figure 3:**
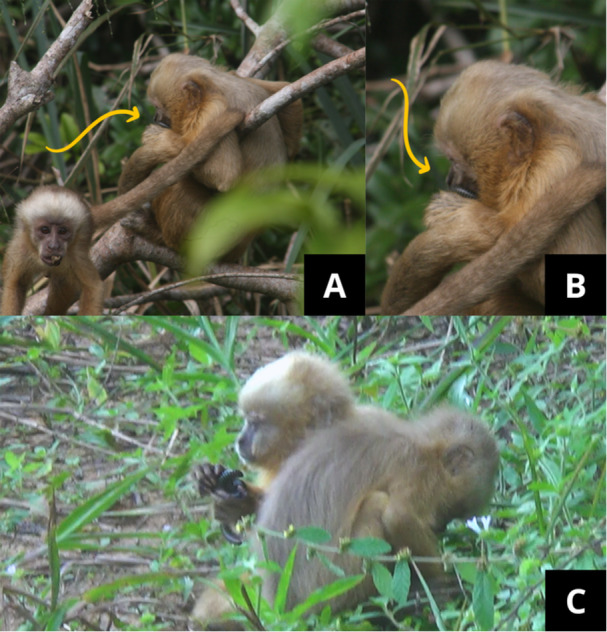
(A) adult male (on the right) and a juvenile (on the left) after a social anointing bout. The juvenile walks away with a millipede in its mouth, and the adult male continues to perform the behavior. (B) Close‐up of the previous image, showing a millipede around the mouth of an adult male. (C) Two juveniles engaged in social anointing on the ground.

The monkeys engaged in anointing with two species of millipedes: *Cladostreptus angustifrons* (Spirostreptida, Spirostreptidae) and *Rhinocricus birivus* (Spirobolida, Rhinocricidae) (Figures 2 and 3). These species, with other members of the orders Julida, Spirobolida, and Spirostreptida, are among the few Helminthomorpha known to produce benzoquinones (Eisner et al. 1978; Weldon et al. 2003; Díaz et al. 2009; Medeiros et al. 2020).

We recorded 12 bouts of social anointing and 22 bouts of solitary anointing. Social bouts were not more frequent (*p* = 0.121) than solitary bouts. The mean duration of solitary bouts was 2.2 min (range: 00:36–7:39 min/s). In social contexts, two or more individuals were observed anointing their own bodies in close proximity, often in physical contact (Figure 3), either using different millipedes or sharing the same individual. The mean duration of social bouts was 4.0 min (range: 01:05–10:06 min/s), although this was not significantly longer than in solitary bouts (*U* = 7, *p* = 0.313).

There were no differences in the rates of anointing performed by adults or juveniles in the general (*χ* ^2^ = 0.24, df = 1, *p* = 0.619), nor in solitary (*χ* ^2^ = 0.77, df = 1, *p* = 0.381) or social context (*χ* ^2^ = 2.13, df = 1, *p* = 0.144). Analyzing only the adults, males anointed more than adult females in general (*χ* ^2^ = 23.97, df = 1, *p* < 0.001), solitary (*χ* ^2^ = 7.14, df = 1, *p* < 0.001), and social context (*χ* ^2^ = 18.54, df = 1, *p* < 0.001).

Overall, anointing occurred more on the inferior strata (*χ* ^2^ = 11.61, df = 3, *p* = 0.008), this was due to a tendency for solitary anointing occurring more on the inferior strata (30 cm–5 m) (*χ* ^2^ = 7.17, df = 3, *p* = 0.054); however, this preference for upper strata was not observed in the social context (*χ* ^2^ = 4.61, df = 3, *p* = 0.159).

**Figure 4:**
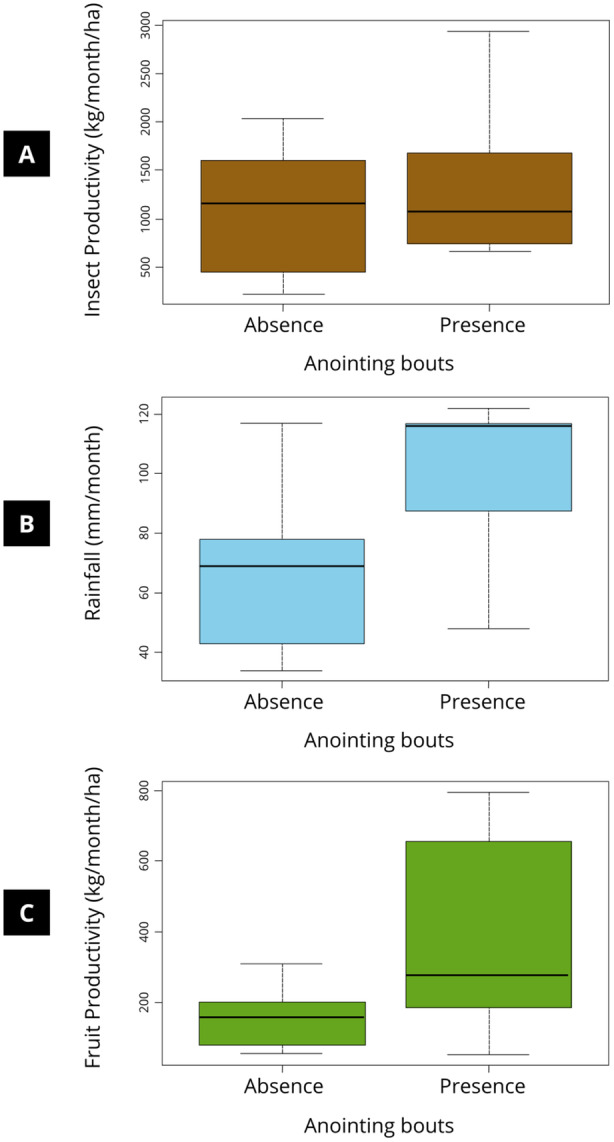
Boxplot of *t*‐test comparing the influence of (A) insect supply, (B) rainfall, and (C) fruit productivity on the presence or absence of anointing in the months.

Anointing bouts did not differ in occurrence between the edge and the interior of the fragment, overall (*χ* ^2^ = 0.015, df = 1, *p* = 0.902), or in solitary (*χ* ^2^ = 0.001, df = 1, *p* = 0.975) and social contexts (*χ* ^2^ = 0.001, df = 1, *p* = 0.973). There was no correlation between arthropod availability and anointing behavior (*U* = −0.85, df = 14, *p* = 0.409; Figure 4A). However, anointing was observed more frequently during midday in general (*χ* ^2^ = 22.94, df = 2, *p* < 0.001), solitary (*χ* ^2^ = 9.51, df = 2, *p* = 0.009) and social context (*χ* ^2^ = 15.29, df = 2, *p* < 0.001), and more bouts occurred during months with the highest rainfall (*U* = −2.69, df = 14, *p* = 0.017) and tended to occur more in periods of higher fruit productivity (*U* = −2.11, df = 14, *p* = 0.053; Figure 4B). It is worth noting that fruit availability and rainfall were positively correlated (rho = 0.54, *p* = 0.034).

## 4 Discussion

### 4.1 Self‐Medication Function Hypothesis

The 34 bouts of anointing analyzed in this study involved two species of millipedes from the orders Spirobolida and Spirostreptida, both known to secrete defensive chemical substances rich in benzoquinones (Eisner et al. 1996; Valderrama et al. 2000; Peckre et al. 2018). These compounds possess antimicrobial properties and act as mosquito repellents, including against *Aedes aegypti* (Weldon et al. 2003). These findings support our prediction (1.1), indicating a self‐medication function, consistent with previous observations of blond capuchins using millipedes in this context (Medeiros et al. 2020).

Our second prediction (i.e., no age–sex differences) was partially supported by our results. No significant differences were found between adults and juveniles, aligning with previous studies showing that this behavior is performed from an early age (Xu et al. 1995; Brodie 1977; Verderane et al. 2007). This supports anointing's potential function in parasite deterrence across age classes. However, a statistically significant difference in anointing behavior was found between adult sexes, with males participating more frequently in anointing bouts than females across all contexts. This finding differs from what was reported by Verderane et al. (2007) and Benítez et al. (2018), who observed no significant difference between age–sex classes. A similar pattern has been documented in black‐handed spider monkeys, where males engaged in more rubbing bouts than females (Laska et al. 2007). This is a novel contribution to the literature by identifying sex as a predictive variable influencing the occurrence of anointing by capuchin monkeys, not only in the general context but also in solitary and social settings.

### 4.2 Social Function Hypothesis

The absence of statistically significant differences between solitary and social anointing bouts in terms of frequency and duration suggests that both contexts may serve overlapping functions, potentially offering similar benefits to the individuals involved. Social anointing, defined here as simultaneous self‐anointing by two or more individuals in physical proximity, and sometimes involving shared use of the same millipede, may serve multiple functions. While our data did not reveal a statistically significant difference between the frequencies of social and solitary anointing, the notably higher number of anointing events by adult males differs from previous reports that described such behavior in *Sapajus* (Verderane et al. 2007; Paukner and Suomi 2008). This discrepancy suggests that, beyond potential medicinal benefits such as ectoparasite deterrence via benzoquinones, anointing in wild populations may also have a social component, such as social communication, as suggested by Laska et al. (2007). Social fur‐rubbing bouts may exert an overall calming and social affiliative effect through the release of oxytocin, as suggested by Benítez et al. (2018). In fragmented landscapes where migration is restricted, and adult males are compelled to remain in their natal areas, this collective release of oxytocin during social fur‐rubbing may facilitate group tolerance. Social functions have been proposed in other primates, including red‐fronted lemurs (*Eulemur rufifrons*) and black‐handed spider monkeys (*A. geoffroyi*), where anointing and rubbing behaviors commonly occur in social contexts (Peckre et al. 2018; Laska et al. 2007).

The greater variability in duration observed in social bouts (range: 01:05–10:06 min/s) may reflect the flexibility of the behavior and its sensitivity to group composition and individual relationships. This extended engagement could also indicate increased motivation or opportunity for social interaction during anointing. Although the sample size limits the strength of statistical inferences, the qualitative differences in the context and bout structure warrant further investigation. These findings suggest that, while self‐medication may be a core function of anointing in capuchins, social contexts may enhance or diversify its role, perhaps functioning simultaneously as a hygienic and social behavior.

### 4.3 Opportunistic Function Hypothesis

Contrary to predictions 3.1 and 3.3, anointing was not observed on the ground and did not occur more frequently at forest edges, where burrowing arthropods are usually found. Additionally, no differences were found across periods of varying arthropod availability. This suggests that millipedes are encountered at similar rates across the fragment, possibly due to the capuchin's ability to visually detect them within the leaf litter. Further research on the species' visual sensory ecology could clarify their ability to detect arthropods across various substrates, including areas with and without leaf litter.

The frequent occurrence of anointing in the inferior strata (off ground but under 5 m), particularly in solitary context but not in social context, may indicate an increased risk of predation during these bouts, potentially due to reduced vigilance while handling and applying the secretions (Peckre et al. 2018). These results suggest social anointing is more spatially flexible, reflecting its potential dual role in self‐medication and social bonding, consistent with Messer et al.'s (2022) results.

In support of predictions 3.2 and 3.4, we observed that anointing was significantly more frequent at midday, coinciding with periods of intense locomotion and foraging activity. This pattern may be related to increased encounters with millipedes during active search for food, as well as greater effectiveness of chemical secretions under higher temperatures. Midday may also offer behavioral opportunities for anointing as individuals pause between foraging bouts.

This behavior was also observed more frequently during the wet months marked by high rainfall levels, a period which promotes surface activity in millipedes due to increased moisture (Dangerfield and Telford 1991; Dangerfield 1998). Medeiros et al. (2020) suggested that the increased number of events during rainy seasons was due to its use as a repellent, since this period coincides with mosquito abundance. However, we cannot discard the opportunistic hypothesis, since this peak may be due to the emergence of millipedes, which also occurs in the rainy season. Fruit availability also coincides with increased pluviometry, and the increased occurrence at midday suggests this behavior occurs at periods when animals are less constrained to find food and move less to avoid midday heat stress. These findings suggest that both ecological availability and daily activity patterns influence the timing of anointing behavior.

We also examined qualitative visual evidence for potential chemically induced states resembling trance, frenzy, or ecstasy in the capuchin monkeys, as previously described in wild red‐faced lemurs (*E. rufifrons*, Peckre et al. 2018). Although we observed repetitive and frenetic movements, during which individuals vigorously rubbed the millipede on their faces, bodies, and arms, no clear signs of trance‐like behaviors were detected, so we cannot attribute a clear recreational function to this behavior.

## 5 Conclusions

Our findings indicate that anointing behavior in blond capuchin monkeys (*S. flavius*) is linked to a self‐medication function, as individuals used millipedes known to secrete chemicals with antimicrobial and repellent properties. The lack of significant differences between adults and juveniles suggests this behavior is useful for all individuals. Nevertheless, anointing was not observed when individuals presented wounds or hair loss, rather it was observed during rainy and more productive seasons, during midday periods, and more often on the lower strata. This suggests this behavior occurs when millipedes emerge from the ground, and during times of high habitat productivity when capuchin monkeys may fulfill their energetic needs more efficiently, allowing them to allocate more time to opportunistic behaviors such as resting, positive, and social interactions. While the frequency of anointing did not differ between social and solitary contexts, the prevalence of adult male participation points to potential secondary functions, such as social communication and calming effects. The increased duration in social relative to solitary bouts, along with qualitative observations of interaction during these events, suggests that anointing may also serve as a mechanism for social bonding or affiliation. Taken together, our results support the idea that anointing is a multifunctional behavior, combining elements of body care, opportunistic use, and social interaction. Future research into the species' sensory and cognitive abilities could shed light on how they locate arthropods in their environment and whether anointing serves communicative and social tolerance purposes within the group.

## Author Contributions

**Ana Paula de Brito‐Araújo:** methodology, writing – review and editing, writing – original draft, data curation, formal analysis. **Natsumi Hamada‐Fearnside:** methodology, writing – review and editing, data curation, formal analysis. **Simone Peruzzo:** methodology. **Italo Ferreira Pereira:** methodology. **Poliana Gabriele Alves de Souza Lins:** methodology. **Kyle Miller:** methodology (geographic data analysis). **Patrícia Elesbão da Silva Rodrigues:** methodology (millipede identification). **Luiz Felipe Moretti Iniesta:** methodology (millipede identification). **Renata Gonçalves Ferreira:** funding acquisition, writing – original draft, writing – review & editing, methodology, data curation, formal analysis, supervision and project administration.
